# Enhanced Originality of Ideas in Women During Ovulation: A Within-Subject Design Study

**DOI:** 10.3389/fpsyg.2022.859108

**Published:** 2022-06-09

**Authors:** Katarzyna Galasinska, Aleksandra Szymkow

**Affiliations:** Center for Research on Biological Basis of Social Behavior, SWPS University of Social Sciences and Humanities, Warsaw, Poland

**Keywords:** ovulatory cycle, sexual selection, creativity, mating behaviors, signaling theory, women

## Abstract

The signaling theory suggests that creativity may have evolved as a signal for mates. Indeed, its aesthetic value might not have been necessary for survival, but it could have helped to attract a mate, fostering childbearing. If we consider creativity as such a signal, we should expect it will be enhanced in the context related to sexual selection. This hypothesis was tested mainly for men. However, both men and women display physical and mental traits that can attract a mate. Previous studies showed that women can be more creative during their peak fertility. We advanced these findings in the present study, applying reliable measures of menstrual cycle phases (examining saliva and urine samples) and the highly recommended within-subject design. We also introduced and tested possible mediators of the effect. We found women’s ideas to be more original during ovulation compared to non-fertile phases of the ovulatory cycle. The results are discussed in the context of signaling theory and alternative explanations are considered.

## Introduction

Men and women both display traits that can attract a mate (Whyte et al., 2021). Men display broad shoulders and facial masculine features. Women display broad hips, breasts, and feminine facial features. Such traits serve as natural ornaments (Miller, 2000a), and as such may indicate the potential partner’s ability to cope with parasites, malnutrition, and social competition, foreshadowing the quality of genes that may be passed on to offspring (Zahavi, 1975; Sugiyama, 2005). However, although this utilitarian Neo-Wallacean view of sexual selection is prevalent in the scientific community, there is a possibility that display traits are not indicating anything, but are merely preferred (Prum, 2012; see also Petrie, 2021). In his book “The descent of men, and selection in relation to sex,” Darwin (1871) proposed that many secondary sexual ornaments are entirely arbitrary and as such do not provide any particular value or utility. Furthermore, Darwin (1871) argued that elaborate displays being the result of sexual selection may also contribute to behavioral abilities.

Indeed, depending on the short- or long-term context of romantic relation, people value specific attributes in a potential mate (Buss and Schmitt, 1993). Attractive traits can belong to various domains (Miller, 1999, 2000a,b), which has been evidenced for the domain of music (Varella et al., 2010; Charlton, 2014; Kaufman et al., 2016; Madison et al., 2018), humor (Kaufman et al., 2008; Greengross and Miller, 2011; Driebe et al., 2021), creativity (Li et al., 2002), and art (Clegg et al., 2011). Creativity definitely has its utilitarian value: it has probably allowed for the development of new ways of enabling survival, such as improving hunting methods or building shelters. As Darwin pointed out (1871, p. 74): “The Imagination is one of the highest prerogatives of man. By this faculty he unites former images and ideas, independently of the will, and thus creates brilliant and novel results.” However, creativity is also strongly associated with aesthetics (Koestler, 1964), manifesting in painting or dancing, which remains unexplained in terms of the need for survival nor daily habits of life (Darwin, 1871). Beauty is not required for survival, and as Darwin (1871, p. 61) argued: “the taste for the beautiful is confined with the attraction of the opposite sex.”

Providing evidence that creativity is an effect of sexual selection is very difficult. However, if we assume such a possibility, we can search for specific consequences. Namely, creativity should be detected and valued by opposite-sex members. Indeed, studies showed that creativity is perceived as sexy by both men and women (Feist, 2001; Li et al., 2002; Kaufman et al., 2016), and is found to be among the top 10 most desired traits worldwide (Buss and Barnes, 1986). Women value creativity in men in the short-term mating context, especially when fertile (Haselton and Miller, 2006; Charlton, 2014). Men prefer females’ ornamental and aesthetic creativity (Kaufman et al., 2016).

For bodily and cognitive traits to evolve by natural or sexual selection, some of the individual variation should have a heritable component (Croston et al., 2015). It seems to be the case with creativity. For example, Waller et al. (1993) reviewed studies on twins and concluded that approximately 22% of variation in creativity (namely divergent thinking) is due to the influence of genes. Also, very recently, Zwir et al. (2022) have provided evidence on the genetic networks underlying human creativity. However, studies are inconclusive on the topic of reproductive success that creativity brings (Clegg et al., 2011; Lebuda et al., 2021). For instance, there is evidence that number and quality of creative works among professionals are positively related to the number of their sexual partners (Clegg et al., 2011), and to the interest they evoke in women (Clegg et al., 2008). At the same time, studies by Lebuda et al. (2021) on non-WEIRD population indicated negative correlations between creative potential and number of living children and grandchildren, as well as between creative potential and the number of spouses.

Creativity as a product of sexual selection should also be enhanced in mating contexts. Griskevicius et al. (2006) showed that creative thinking in men can be enhanced in the context of any potential partner, but for women a high-quality and committed partner is needed. The periovulatory time can also provide such a context for women, as it is the only period when sex can result in conception (Gildersleeve et al., 2014). This physiological process involves the release of the dominant ovarian follicle from the ovary into the fallopian tube, where it can be fertilized (Holesh et al., 2021). This moment should activate mechanisms involved in the process of sexual selection not only on a physiological, but also on a psychological and behavioral level. Indeed, throughout the cycle, women are supposed to experience adaptive changes in their subconscious mental and behavioral processes associated with mating (Gangestad and Thornhill, 1998, 2008). They are more sexually aroused (Roney and Simmons, 2013) and interested in mating concerning extra-pair copulation (Gangestad et al., 2005; Pillsworth and Haselton, 2006) or primary partner (Pillsworth et al., 2004). Few studies point to general increase in sexual desire (Jones et al., 2018). Furthermore, arousal seems to manifest itself in domains not associated with sex, such as increased motoric activity (Udry and Morris, 1970), found at the physiological level (Gómez-Amor et al., 1990; Krug et al., 1996).

The elevated self-promotion found in women during increased fertility can be considered a prominent signal manifestation. Women care for their appearance significantly more, trying to attract a potential partner with self-ornamentation, a more fashionable style (Haselton et al., 2007), and revealing clothes (Durante et al., 2008). However, it must be noted that these behaviors can serve not only intersexual selection, but also intrasexual selection (Fisher et al., 2009). Moreover, studies report that women during the fertile window of the ovulatory cycle are more determined to meet potential mates by attending social gatherings (Haselton and Miller, 2006). Some authors suggest that due to such actively realized mating goal, they eat less (Fessler, 2003; Roney and Simmons, 2017) and are more prone to risky behaviors (Šukolová and Sarmány-Schuller, 2011), which is linked to decreased cognitive control found in women during the fertility peak (Hatta and Nagaya, 2009).

It appears that ovulation is a condition dictated by special needs that women signal. Furthermore, their signals are received. Studies show that women are evaluated as more attractive precisely during the fertility peak (Roberts et al., 2004). Men score women’s facial appearance as better (Roberts et al., 2004; Puts et al., 2013) and the same goes for their vocal pitch (Pipitone and Gallup Jr., 2008; Puts et al., 2013), and body scent (Singh and Bronstad, 2001; Gildersleeve et al., 2012). As psychological traits can also be attractive, these kinds of signals should be manifested by women as well. However, there is hardly any research to show that. Varella et al. (2017) discuss the role of female ornamentation as overlooked ancestral selective pressure in the evolution of artistic propensities. Previous studies focused mainly on men, investigating how they may change their behavioral manifestation to attract a potential mate (Tifferet et al., 2012; Gao et al., 2017; Bongard et al., 2019). This is probably because, due to parental investment theory (Trivers, 1972), men are supposed to be a less investing sex, and hence, less choosy about a potential partner. In this view, men must court their female partners more, performing more signaling. However, although true in case of many sexually dimorphic species (Janicke et al., 2016), this view does not fit well with quite sexually monomorphic humans (Miller, 2013; Stewart-Williams and Thomas, 2013). Both women and men are highly selective, especially when it comes to long-term relationships (Buss and Barnes, 1986; Lippa, 2007). It has been suggested and shown that mutual ornaments can have a signaling function in both sexes (Kraaijeveld et al., 2007), and as physical attractiveness is highly important for female mate value, it could be argued that women should prevail not only in the aesthetic domain, but in creative artistic domains in general (Varella et al., 2017). This view has been extensively confirmed by Varella et al. (2017). who reviewed evidence indicating that women are more likely than men to involve artistry in the contexts of inter- and intrasexual selection.

In our studies, we verify hypotheses concerning creativity as a sexually selected trait in women. If we assume that, we should expect it will be enhanced during the fertile phase of the ovulatory cycle, when conception can occur (Gildersleeve et al., 2014). The results of our previous study (Galasinska and Szymkow, 2021) indicate that women’s creativity may increase with their fertility. These results are consistent with the signaling theory (Miller, 2000a) and replicate the previous studies conducted by Krug et al. (1994, 1996). We have found that as the probability of conception gets higher, women’s thinking becomes more divergent. Divergent thinking leads the individual to numerous and varied responses, being commonly used as an estimate of creative potential (Runco, 2007). This kind of thinking concerns three dimensions: fluency, flexibility, and originality (Guilford, 1968). Among them, it is originality that is the most critical indicator and the primary facet of creativity (Acar et al., 2017). Originality refers to things that are novel and slightly different from others (Lugo et al., 2016). In our study (Galasinska and Szymkow, 2021), two of three dimensions of divergent thinking, namely originality and flexibility, were positively correlated with the probability of conception, and the effect of originality was the strongest.

We also introduced arousal as a mediator, as arousal is found to be increased during peak fertility (Gómez-Amor et al., 1990; Krug et al., 1996; Roney and Simmons, 2013). Arousal is one of the core features of emotional response to environmental challenges with amplified motivation toward the readiness to act (Clore and Storbeck, 2006). It reflects the intensity of behavior, referring to the degree of excitation, activation, and energy mobilization (Duffy, 1962). Activating moods are found to facilitate divergent thinking (Baas et al., 2008). If the mediation of arousal in our study was significant, it would point to an important conclusion that creativity may be promoted as a side effect of increased arousal, which itself can be an adaptation of the way of finding a partner. Such an effect would undermine legitimacy of the signaling role of creativity in this context. However, we found no mediating effect of self-reported arousal. Increased arousal during ovulation may be the effect of increased dopamine release during this phase (Colzato et al., 2010), which is found to improve both divergent thinking and mood (Ashby et al., 1999). Due to this fact, in the present study we decided to additionally test the role of mood, as its facilitating role in creativity is well studied (Fredrickson, 2004; Baas et al., 2008; De Dreu et al., 2008), and cognitive control, associated with flexibility (Isen et al., 1987).

### Present Study

This study aimed at replicating the previous one (Galasinska and Szymkow, 2021), in a within-subject design, recommended in studies considering the ovulatory cycle (Gangestad et al., 2016), and importantly, with the use of more reliable measures of cycle phases (examining saliva and urine samples). We hypothesize that ovulating women will be more fluent and flexible in thinking, and more original in ideas comparing to those in non-fertile phases. Furthermore, we hypothesize that they will be more aroused, in a higher mood, and having lower cognitive control comparing to other phases. We will check whether these variables will mediate the effect of enhanced creativity. The original contributions presented in the study are publicly available. This data can be found at https://osf.io/6ypkb/.

## Materials and Methods

### Participants

With the use of Sona system at the SWPS University of Social Sciences and Humanities, we recruited 94 Polish women in reproductive age (19–35), cycling naturally from 21 to 35 days. We excluded 22 participants due to reasons presented in Supplementary Figure 2. The final sample comprised 72 women (*M_age_* = 25.53, *SD_age_* = 5.05), its size was estimated in accordance with Gangestad et al. (2016) recommendations for within-subject designs. The participants reported not using hormonal contraceptives, not being pregnant, breast feeding, nor having given birth for at least 3 months prior to study participation. The frequency analysis revealed that 39 participants reported being in a relationship lasting from 2 months to 16 years (*M_years_* = 2.72, *SD_years_* = 3.69), one participant reported being homosexual, and 10 being bisexual. Also, three participants reported taking antidepressants. We collected the data between July 2020 and April 2021, during the COVID-19 pandemic, a time of relative social isolation. People were predominantly working from home and public social life was suspended due to government sanitary restrictions.

### Materials and Procedure

#### Menstrual Cycle Phase Determination

We studied women during three menstrual cycle phases: early follicular (menstrual), ovulatory, and late luteal (premenstrual), always keeping a minimum of 1-week interval between measurements. The sequence of phases was randomized: the order of the three phases was drawn for each participant before the study began, so each participant had a different order of phases. We present the size of the groups starting the study in each phase in Supplementary Figure 1. Following the report by Su et al. (2017), we applied a saliva-based method using ovulatory microscopes (Geratherm) to confirm accuracy of the phases, and urine LH test kits to confirm the results of the microscopy. Study participants were given microscopes and LH tests *via* post and were instructed with a tutorial film on how to use them properly.

#### Creative Divergent Thinking

Divergent thinking involves fluency (production of ideas), flexibility (their variety), and originality (their uniqueness), which are general factors of creative potential, i.e., can result in creative products of any kind (Runco, 2007). The most commonly used estimate for such creative potential are open-ended alternate uses tasks (Benedek et al., 2014). In our study, we administered a computerized version of the Alternative Uses Test (AUT; Guilford, 1967). Participants were given a name of a common object and were asked to generate and write different unusual and creative uses for that object in a 5-min period. One of three common objects was applied in each phase, in randomized order: a shoe, a towel, and a bottle. Participants’ ideas were scored based on fluency, flexibility, and originality by four trained, independent raters (psychology students), blind to hypothesis, and participants’ cycle phases. Raters were tested for inter-rater reliability. *Fluency* scores were the sum of ideas provided. *Flexibility* was assessed by the number of semantic categories applied (Runco, 2007). Each rater had to indicate and collect categories as they saw fit, for example: “art-related,” “weapon-related,” or “construction related.” However, the breadth of categories was determined by each rater’s individual characteristics. Averaged *originality* was rated on a 5-point scale (from 1 = *not original* to 5 = *highly original*). The total originality score was divided by the number of ideas to prevent a confounding effect of fluency (Runco and Acar, 2012; Forthmann et al., 2020).

#### Creative Convergent Thinking

Another paradigm of creativity suggests that it takes distant associative abilities to identify the best matching idea as a solution to a problem (Wu et al., 2020). To test this paradigm of creativity, we applied Remote Associates Test (RAT; Mednick, 1968; Polish version by Sobków et al., 2016), a convergent thinking test (Lee and Therriault, 2013) significantly related to insight problem solving (Chang et al., 2016). It consists of 17 sets of three words that are associated with the fourth word (the solution). We applied 15 items, divided into three phases. Sets of five items per phase were randomly selected for each participant. All item sets are presented in Supplementary Material.

#### Arousal and Its Valence

To examine the general arousal and its pleasantness reported by participants, we applied the Self-Assessment Manikin (SAM; Bradley and Lang, 1994), a picture-oriented survey measuring emotional response. We used the subscale of valence/pleasure (five pictures ranging from the most negative = 1 to the most positive = 5) and arousal (rated from low = 1 to high = 5).^1^ The participants were asked to match the picture that corresponded with their state.

#### Mood

We administered the Mood Adjective Checklist (UMACL; Matthews et al., 1990) adapted to Polish by Goryńska (2005), consisting of 29 adjectives describing emotions. Participants used a 4-point scale (from 1 = *definitely not* to 4 = *definitely yes*) to rate if they experienced a particular emotion at the moment of rating. We averaged scores for the three subscales: energetic arousal, tense arousal, and hedonic tone (all *α* ≥ 0.90).

#### Cognitive Control

We administered a color-word Stroop task, with the use of Inquisit Lab programme, drawing from the on-line test library of the Millisecond platform. Participants were asked to install the program on their computer and practice it two times. Contact email address and phone number were provided in case of any questions. The task assesses the ability to inhibit cognitive interference occurring when processing of the stimulus feature affects the simultaneous processing of the other attribute of this stimulus (Stroop, 1935). The instruction was to categorize the color of presented latter strings as either red, green, blue, or black, using the *d*, *f*, *j*, or *k* key, respectively, ignoring the meaning of the word suggesting the color. The background was white. The response mappings (*d*, *f*, *j*, *k*) were continuously displayed. Error responses were followed by a 1,000-ms visual error message (X) and were excluded from further analysis. The task consisted of 84 consecutive trials of randomly presented congruent, incongruent, and control stimuli (28 stimuli per category). Stroop interference was assessed by subtracting the reaction time to incongruent and congruent stimuli. We had to exclude participants with incomplete trials (*N* = 15) from further analysis of results of this task.

#### Procedure

Continuous email and phone contact with each subject was maintained, as the COVID-19 pandemic has halted laboratory studies. Women were informed they would be participating in a study on mental associations across the menstrual cycle. Qualified participants were given a consent form and indicated the address where study materials were to be sent. They received a tutorial film on the microscope and LH test kits use, and a step-by-step diagram with the images of potential microscope results in each phase. Participants then completed the initial online demographic survey, including the training for the Stroop task. After getting the materials, participants were called and had a chance to ask questions. The instructions were discussed once again to ensure women understood them properly. Participants reported the first day of their last menstruation and previous cycles’ length and were instructed to monitor their cycle every morning, using a microscope. They were also asked to provide an index of microscope results for each day, marking it in the calendar together with the first day of the following menstruation. We informed them that they should expect to ovulate between days 10 and 17 after menstruation (Holesh et al., 2021 +/− 4 days’ deviation). To read the microscope result, they compared the view with three potential result images provided by the microscope manufacturer.

To test for the follicular phase, women reported the onset of menstruation and appropriate microscope image. They got the link to the survey on the second or third day, to avoid painful symptoms that may accompany the onset of menstruation (Krug et al., 1994). They were asked to inform the experimenter if painful symptoms were salient. If so, the examination time was to be postponed until the following day. To test for the ovulation phase, women reported the appropriate image from the microscope. To confirm ovulation detected by microscope result, they conducted a urine LH test. They sent a picture of LH test kit result to the experimenter, who verified whether it indicated ovulation or not. If not, from that day, the procedure of testing for ovulation with both types of tests was repeated every day until the experimenter assessed the results of testing as positive. If ovulation was not confirmed by both tests until the 17th day of the cycle (Holesh et al., 2021 +/− 4 days’ deviation), the whole procedure was repeated in the following ovulatory cycle. If it was confirmed, women were given a survey link. We did not test for creativity unless two tests positively and consistently indicated ovulation. To test for the luteal phase, women reported the 20th day of the cycle and negative result of the microscope test. Survey link was sent to them within a few days, but not earlier than 1 week after the previous measurement. We present the detailed procedure diagram in Supplementary Figure 2.

In each phase of the survey, the participants indicated their subjective feeling of arousal and mood (SAM), they marked on the scale how strongly they felt each emotion at the time (UMACL), they performed the Stroop task, and listed unusual uses of the object named on the screen, within a 5-min period (AUT). The following day they completed another survey, testing creative convergent thinking. The survey consisted of marking arousal and valence (SAM), indicating experienced emotions (UMACL), and performing a creative convergent thinking task (RAT), in which participants were asked to find a word associated with three other words shown on the screen within 30 s.

## Results

Using IBM SPSS Statistics 25, we first calculated descriptive statistics for all studied variables, both with the Shapiro–Wilk test. Despite the disturbed normality of all variable distributions, a distribution for the vast majority of variables, specifically for these which were expected to replicate the effect of the previous study, was found to be asymmetric to a small extent, as a skewness of it did not exceed a conventional absolute value of 2. Therefore, we assumed it was reasonable to carry out the analysis based on parametric tests. We administered the Repeated Measures Analysis of Variance ANOVA with post-hoc Scheffe test comparisons for homogenous groups, and Games–Howell test comparisons for others. We applied t-tests for dependent samples to compare combined infertile phases to the fertile phase of ovulation. We calculated partial eta squared to measure the effect size in ANOVA models, and Cohen’s *d* in case of t-test comparisons. To correct for multiple testing, we used the Bonferroni correction. Specifically, we used the value of 0.017 as the critical significance level for the comparisons of three phases, and 0.025 for the comparisons of fertile to non-fertile phases. We calculated Kendall *W* for the inter-rater reliability of creative divergent thinking assessments. To test the hypothesized role of potential mediators, we applied the MEMORE macro from SPSS, which allowed to infer about indirect effects based on 5,000 bootstrap samples in repeated measures (Montoya and Hayes, 2017).

### Creative Divergent Thinking

Kendall *W* for the inter-rater reliability of the divergent thinking scores for the phase of ovulation was: fluency *W* = 0.89, *p* < 0.001, flexibility *W* = 0.65, *p* < 0.001, originality *W* = 0.56, *p* < 0.001. For the follicular phase: fluency *W* = 0.98, *p* < 0.001, flexibility *W* = 0.70, *p* < 0.001, originality *W* = 0.52, *p* < 0.001. For the luteal phase: fluency *W* = 0.77, *p* < 0.001, flexibility *W* = 0.70, *p* < 0.001, originality *W* = 0.63, *p* < 0.001. These results indicated strong raters’ cohesion (Moslem et al., 2019).

**Figure 1:**
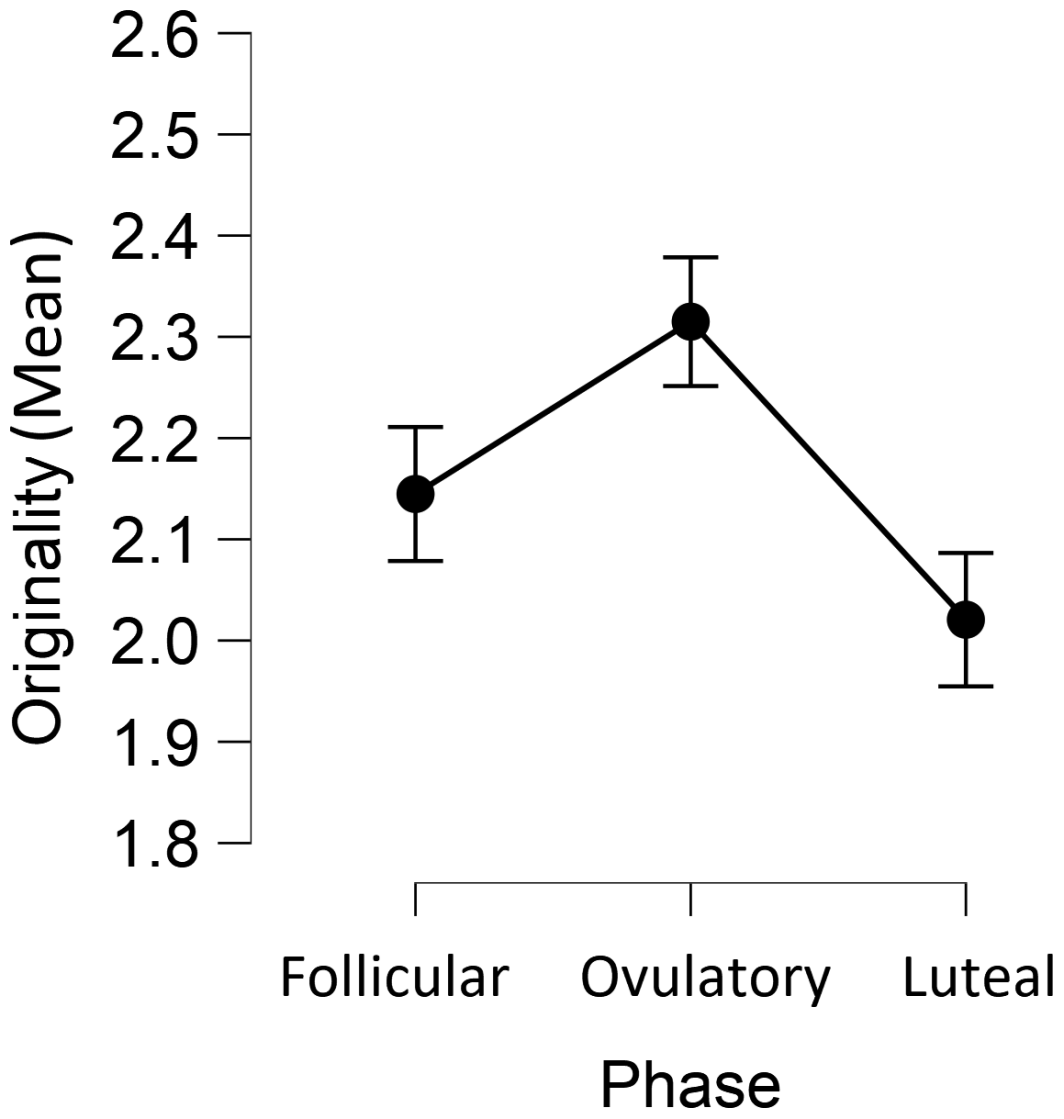
Mean scores of originality of ideas in each phase of the ovulatory cycle.

As shown in Figure 1 and Table 1, participant’s ideas were the most original during the phase of ovulation *F*(2, 142) = 8.99, *p* < 0.001, *η_p_*^2^ = 0.11, and the least original during the late luteal phase. Flexibility of thinking was not differentiated between phases, *F*(2, 142) = 1.65, *p* = 0.195, *η_p_*^2^ = 0.02, nor was the fluency, *F* < 1.

**Table 1:** Differences between ovulatory cycle phases in terms of variables studied.

	Follicular		Ovulation		Luteal		*F*	*p*	*η* ^2^
	*M*	*SD*	*M*	*SD*	*M*	*SD*			
Fluency	11.49	6.14	11.57	5.97	11.79	5.56	0.17	0.847	0.00
Flexibility	4.43	1.68	4.73	1.75	4.56	1.57	1.65	0.195	0.02
Originality	2.14	0.56	2.32	0.54	2.02	0.56	8.99	**<0.001**	0.11
Creative convergent thinking	2.01	1.69	2.04	1.61	1.97	1.76	0.04	0.959	0.00
Arousal	2.78	0.98	3.22	0.97	2.79	1.01	4.75	**0.010**	0.06
Valence	3.35	1.14	3.90	0.92	3.68	0.95	5.76	**0.004**	0.08
Energetic arousal	2.67	0.73	3.11	0.68	3.42	0.83	19.07	**<0.001**	0.21
Tense arousal	1.87	0.65	1.71	0.61	2.01	0.36	7.45	**<0.001**	0.10
Hedonic tone	2.79	0.77	3.16	0.65	3.00	0.76	5.56	**0.005**	0.07
Cognitive control	152.97	146.89	170.89	154.18	173.82	152.98	0.33	0.722	0.01

M, mean; SD, standard deviation; F, Anova coefficient; *p, level of significance;* and η^2^, partial eta squared effect size.

As the literature on the ovulatory cycle often reports fertility as a binary categorical variable, we combined the results of divergent thinking from the infertile phases and compared them to the divergent thinking results from the fertile phase. We found originality to be higher during the fertile (*M* = 2.32, *SD* = 0.54) compared to non-fertile phases (*M* = 2.08, *SD* = 0.49), *t*(71) = 3.56, *p* < 0.001, *d* = 0.42. The mean for flexibility was higher in the fertile phase (*M* = 4.73, *SD* = 1.75), comparing to non-fertile ones (*M* = 4.49, *SD* = 1.43). However, as we corrected for multiple testing, this effect reached significance only for one-tailed test, *t*(71) = 1.93, *p* = 0.014 (one-tailed), *d* = 0.23. No differences in fluency were found, *t*(71) = 0.16, *p* = 0.874. *d* = 0.02.

### Creative Convergent Thinking

We found no differences in convergent creative thinking, *F* < 1.

### Arousal and Its Valence

Participants’ general arousal during ovulation was higher compared to other phases, *F*(2, 142) = 4.75, *p* = 0.010, *η_p_*^2^ = 0.06, with no differences among the other two phases (*p* = 0.996). Its valence was also the most positive during ovulation, *F*(2, 142) = 5.76, *p* = 0.004, *η_p_*^2^ = 0.08 (see Table 1).

### Mood

Hedonic tone was differentiated between phases, *F*(2, 142) = 5.56, *p* = 0.005, *η_p_*^2^ = 0.07. It was significantly higher during ovulation comparing to the follicular (*p* = 0.011), but not to the luteal phase (*p* = 0.424), with no difference between non-fertile phases (*p* = 0.233). The energetic dimension was also differentiated, *F*(2, 142) = 19.07, *p* = 0.001, *η_p_*^2^ = 0.21. It was higher during luteal compared to follicular phase (*p* < 0.001), but not significantly different compared to ovulation (*p* = 0.042). During ovulation, it was higher compared to follicular phase (*p* < 0.001). Tense dimension was differentiated, *F*(2, 142) = 7.45, *p* < 0.001, *η_p_*^2^ = 0.10. It was similar during the follicular and luteal phases (*p* = 0.248). It was significantly lower during ovulation compared to luteal (*p* < 0.001) but not to follicular phase (*p* = 0.297; see Table 1).

### Cognitive Control

We found no differences in cognitive control, *F* < 1.

### Arousal as a Mediator

To perform mediation analysis, we aggregated data regarding follicular and luteal arousal as infertile phases arousal, and ovulation as fertile phase arousal. Direct effect of originality *b* = 0.23 was significant, as zero fell outside the appropriate 0.95 interval [0.102, 0.363]. Direct effect of arousal was also significant *b* = 0.44, 95% CI[0.169, 0.706]. However, the analysis revealed no indirect (mediating) effect of repeatedly measured arousal, *b* = −0.01, 95% CI[−0.054, 0.040]. Detailed results in Table 2.

**Table 2:** Effect coefficients of the analysis testing arousal and mood as mediators of originality of ideas.

	Arousal				Mood			
	*b*	*SE*	CI 95%^*^		*b*	*SE*	CI 95%^*^	
			*LL*	*UL*			*LL*	*UL*
Ydiff	0.23	0.07	0.102	0.363	0.23	0.07	0.102	0.363
Mdiff	0.44	0.13	0.169	0.706	0.39	0.13	0.136	0.642
Direct	0.24	0.07	0.101	0.382	0.23	0.07	0.092	0.372
Indirect	−0.01	0.02	−0.057	0.039	0.00	0.03	−0.055	0.059

* 95% CI is presented as bias-corrected and accelerated 5,000 bootstrapping.

### Positive Mood as a Mediator

We aggregated data on follicular and luteal valence as infertile phases valence, and ovulation as fertile phase valence. Direct effect of valence was significant, *b* = 0.27, 95% CI[0.087, 0.041], however indirect (mediating) effect was not, *b* = −0.01, 95% CI[−0.259, 0.796]. To confirm this effect, we conducted the same analysis using hedonic tone as a mediator. Direct effect of hedonic tone was significant, *b* = 0.37, 95% CI[0.153, 0.583], but the indirect (mediating) effect was not, *b* = −0.01, 95% CI[−0.070, 0.056]. Detailed results in Table 2.

## Discussion

The aim of the research was to replicate the study investigating enhanced creative potential of fertile women, with the use of more reliable measures of the phases, and more appropriate within-subject design. We tested women during follicular, ovulation, and luteal phases, hypothesizing to find the effect during ovulation. Our hypotheses were based on the signaling theory (Miller, 2000a), which states that creativity may have evolved as a signal for mates. Although we cannot confirm its role as an indicator of fitness, our study suggests that it may be a mental ornament in women, related to the process of sexual selection (Darwin, 1871). Such an ornament should be manifested in the contexts associated with mating, like, for example, during a fertile phase of the ovulatory cycle.

In our study, originality of ideas was enhanced among fertile women. Originality is called an impression stimulator (Runco, 2007), as it affects attention. This sort of saliency starts at the sensory level (Gaspelin and Luck, 2018). As the most captivating feature of creativity, originality is also found to be the strongest predictor of it (Diedrich et al., 2015). There are also various ways to achieve original ideas. Flexibility of thinking can lead to such ideas through breaking patterns (Runco, 2007). In our study, flexibility was not differentiated in the comparison of three phases. But, it was higher during fertile phase, compared to less fertile phases combined. Different processes may also foster originality, for example persistence (Nijstad et al., 2010). Further studies are needed to test this idea. The fluency dimension was not differentiated either. The probability to generate an original idea increases with the number of ideas. However, the number of ideas is not essential, as a creative person may produce only one idea, but it may be an original one (Acar et al., 2017). Women had a similar quantity, but different quality of ideas. Furthermore, this quantity was quite high in each phase (about 11 ideas on average per phase), so we can assume that participants were generally motivated to produce ideas in the study. We cannot exclude the influence of the pandemic, as partial isolation might have affected participants’ willingness to engage in any kind of activities related to the outside world, creative activities in particular (Karwowski et al., 2021). This generic increased motivation may have also influenced diversity of their thoughts (flexibility), as this dimension was also not differentiated between phases. However, such motivation was not sufficient to produce similarly original ideas in each phase. Thus, it is difficult to interpret differences in originality between phases in the context of isolation, as it was a fixed condition across the phases. Female’s fertility and cycle length are considered to be affected due to illness (Carp-Veliscu et al., 2022) or vaccination (Nguyen et al., 2021). However, the study was conducted in the pre-vaccine (for COVID-19) period. None of the screened participants reported being sick. Morbidity rates during that time were relatively low when we compare them to the following years. However, we cannot exclude asymptomatic cases of COVID-19. We want to emphasize that we did not investigate creativity in participants whose ovulatory cycle was disturbed. The length of all screened cycles was differentiated within a range from 27 to 35 days, so we did not observe notable changes in the cycle length, in the cases when ovulation normally occurred.

Miller (2000a) outlines that creativity, as a subject of selection, concerns a domain associated with aesthetics and fine arts rather than technological innovation. Darwin (1871) pointed to a ‘sense of beauty,’ suggesting a mechanism for mere aesthetics with no direct benefits. Wallace, on the contrary, pointed to the good-gene, utilitarian model, suggesting signals of vigor and vitality behind the signals of beauty, which started a debate on how exactly the mechanisms of sexual and natural selection interact (Prum, 2012; Hoquet and Levandowsky, 2015). Creative ideas are domain-general and defined as novel and useful (Runco, 2007). However, studies indicate that the effect of novelty is larger than usefulness and the latter is not necessarily predictive of creativity (Diedrich et al., 2015). It is also hard to miss the difference between technology and fine arts. The common variance of creativity and intelligence is found to be moderate, and researchers outline the orthogonality of these two constructs (Runco, 2007). Technological creativity would more likely fit the Wallacean utilitarian view of sexual selection processes (Feist, 2001). As divergent and original thinking is assumed to be independent of IQ (Wallach and Kogan, 1965), in our study we have additionally involved a creative convergent thinking test, reflecting the correlation of creativity and intelligence (Lee et al., 2014), and hence more relevant to survival problem solving. Eventually, we found no differences in these abilities between phases. It leads us to an interesting conclusion, corresponding to the problem of utility or/and beauty aspects of sexual selection. Namely, it is possible that convergent creativity could rather be attributed to natural selection processes, while divergent creativity to sexual selection understood after Darwin (1871) as a non-utilitarian, merely aesthetic mechanism of evolution. Thus, our study suggests that two different types of creativity might have evolved, each one focused on solving problems in different domains, namely survival and reproduction. If so, we should expect divergent creativity, but not the convergent one, to be enhanced in the mating context. This is to be verified in future studies.

The significant role of possible mediators would suggest that creativity may be a by-product of another selection. We tested arousal and positive mood, as they can facilitate creativity (Baas et al., 2008). Men could choose women who were more aroused, or more joyful, not directly creative. Creativity, as facilitated by elevated and activating moods, could have developed in parallel. However, although we found these variables increased during the fertile phase (*vs* infertile phases), we did not detect any mediating effects. Furthermore, both energetic and tense dimensions of mood were the highest during the luteal phase. However, being asked about general arousal, women reported it to be lower comparing to ovulation. We can suppose that during the luteal phase, women experienced mixed emotions. Progesterone may be associated with PMS syndrome (van Wingen et al., 2008), which we did not control unfortunately. But, as estrogen and progesterone act together during the luteal phase, we cannot exclude their interaction in affecting mood in the way we observed. It is important to note, that we awaited the LH peak during ovulation in our study, which usually co-occurs with a pending decrease of estrogen (Reed and Carr, 2018). Direct hormonal measures are needed to explain the mood effects we obtained.

Fertile phase arousal may manifest differently: as a general arousal on the physiological level, but also as mental, sexual, or motoric stimulation, or even as a motivational boost. It is possible, therefore, that the measures we administered might have not been precise enough and they should be more diversified in future studies. We did not control for premenstrual syndrome, which can also be a confounding variable. Finally, we did not control for typing speed (Forthmann et al., 2017), nor the time of day (Breslin, 2019).^2^

We did not find any differences in cognitive control between the phases; however, this result should be taken with caution. The conditions were not standardized, as the study procedure was conducted *via* the Internet. Participants’ PC monitors may differ in size and contrast. Additionally, we were not able to check if all participants did the training as we recommended.

To sum up, the present study replicated the effect of enhanced originality of ideas among women during ovulation (Galasinska and Szymkow, 2021). It suggests that originality in divergent creativity is a plausible candidate for mental ornamentation in women. Being boosted during the fertile phase of the cycle, originality presumably increases mate attraction, potentially leading to conception. Nevertheless, it may also promote intrasexual competition to discourage competitors. More contexts should be studied to confirm the hypothesis on the signaling role of creativity. We presented just one of them, showing that with no other incentives, women may manifest some signals of creativity, which may point to its evolutionary legacy.

## Data Availability Statement

The datasets presented in this study can be found in online repositories. The names of the repository/repositories and accession number(s) can be found in the article/Supplementary Material.

## Ethics Statement

The studies involving human participants were reviewed and approved by the Ethical Review Board of the Faculty of Psychology in Sopot at SWPS University of Social Sciences and Humanities, Poland. The patients/participants provided their written informed consent to participate in this study.

## Author Contributions

KG and AS contributed to the conception of the study and study design. KG conducted the study, organized the database, performed the statistical analysis, and wrote the first draft of the manuscript. AS revised the manuscript critically for important intellectual content. All authors contributed to the article and approved the submitted version.

## Conflict of Interest

The authors declare that the research was conducted in the absence of any commercial or financial relationships that could be construed as a potential conflict of interest.

## Publisher’s Note

All claims expressed in this article are solely those of the authors and do not necessarily represent those of their affiliated organizations, or those of the publisher, the editors and the reviewers. Any product that may be evaluated in this article, or claim that may be made by its manufacturer, is not guaranteed or endorsed by the publisher.

## Footnotes

^1^This tool also includes the third subscale of dominance. In our study, we focused merely on the potential mediating role of arousal and emotions, and thus, we did not investigate the role of dominance. However, as dominance can be important in the context of mating (Stanton and Schultheiss, 2009), and in the context of creativity (Heinzen et al., 1988), we decided to conduct mediational analyses for the dominance dimension as well. No mediating effect was found.

^2^Breslin (2019) found that time of day may diversify creative outcomes, with higher effects before noon. Our participants filled out surveys before noon in the vast majority of cases (during the follicular phase, the ratio was 46 women before noon and 25 after noon; during ovulation: 40 before noon, 28 after noon, and during the luteal phase: 44 before noon and 28 after noon). However, the Breslin’s effect corresponded particularly with the fluency dimension, which was not differentiated between phases in our study.
