# Supplementary material for: Enhanced Originality of Ideas in Women During Ovulation: A Within-Subject Design Study
Source: Front Psychol. 2022 Jun 9;13:859108. doi: 10.3389/fpsyg.2022.859108 (PMC9222335; doi:10.3389/fpsyg.2022.859108)
Supplement: Supplementary file 1 [file Data_Sheet_1.docx]

Supplementary Material

**The Polish version of RAT’s item sets, applied in the study:**

SET 1

Aktor, szeryf, noc

Brew, broń, architektura

Dama, kredyt, menu

Drzewo, ząb, król

Drzwi, rycerz, spodnie

SET 2

Grzebień, korzeń, mądrość

Komiks, wdowa, sieć

Oko, lody, uchwyt

Owoc, kolor, wybuch

Sygnał, stop, pokój

SET 3

Telefon, kucharz, biblia

Telefon, spiżarnia, jądro

Urodziny, ogień, W-F

Więzienie, opakowanie, błąd

Zamek, odlot, pięciolinia


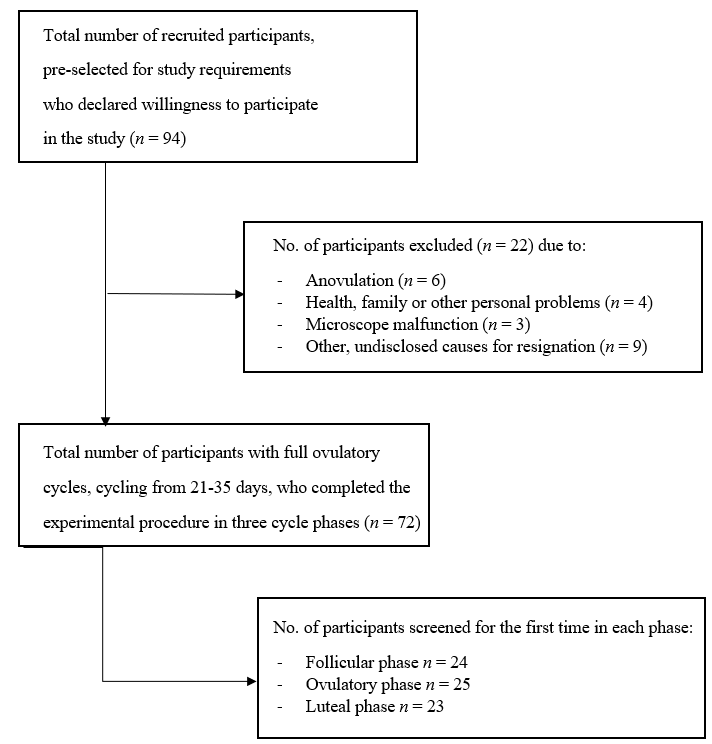


*Figure 1.* Total number of participants recruited, ineligible, and screened, with the indication of the randomly selected phase of the first measurement.


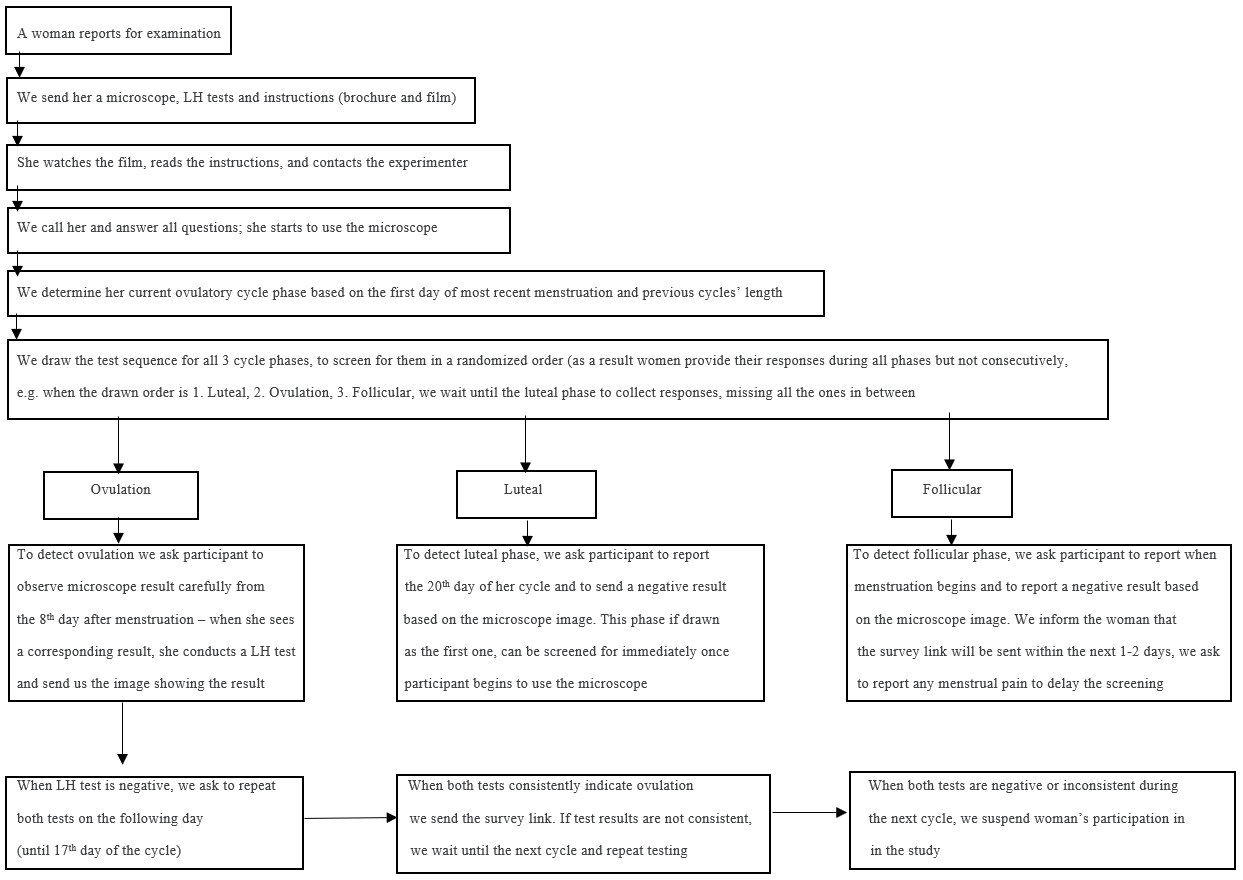


*Figure 2*. Diagram presenting the procedure of testing for each phase of an ovulatory cycle
